# The Use of Technology to Deliver In-Home Aged Care Services: Mixed Methods Study of Australian Staff Perspectives

**DOI:** 10.2196/76141

**Published:** 2025-10-14

**Authors:** Baldwin Pok Man Kwan, Marissa Dickins, Sue Williams, Frances Batchelor, Kerry Hwang, Kate Fulford, Tony Walsh, Helena Jakupovic, Tanya E Davison

**Affiliations:** 1 Research and Innovation, Silverchain, 6 Sundercombe Street, Osborne Park, Western Australia, 6017, Australia, 61 1300650803; 2 School of Public Health and Preventive Medicine, Faculty of Medicine, Nursing and Health Sciences, Monash University, Clayton, Victoria, Australia; 3 Institute of Health and Wellbeing, Federation University, Ballarat, Victoria, Australia; 4 National Ageing Research Institute, Parkville, Victoria, Australia; 5 Department of Physiotherapy, The University of Melbourne, Parkville, Victoria, Australia; 6 School of Nursing and Midwifery, Monash University, Clayton, Victoria, Australia

**Keywords:** aged, digitally enabled care, home care services, technology, workforce

## Abstract

**Background:**

With a global aging population, technology has been proposed as a solution to address the growing demand for services in the in-home aged care sector. Despite the potential of technology, there are difficulties when implementing technology into routine care delivery. There is a lack of evidence regarding the specific factors affecting technology use in the in-home aged care setting from the perspective of the direct care workforce.

**Objective:**

This study aimed to understand in-home aged care staff members’ views of (1) the digital enablement potential of direct in-home care tasks, (2) benefits and drawbacks of technology use, and (3) enablers and barriers for technology use in Australian in-home aged care.

**Methods:**

An explanatory sequential mixed methods research design was used, with a cross-sectional survey and semistructured staff interviews. Participants were recruited from in-home aged care staff members working at a national Australian in-home health and aged care organization.

**Results:**

In total, 226 participants completed the survey, and 18 participants completed the interviews. Overall, participants felt that many care tasks within in-home aged care could be digitally enabled, with more than half (56%) of the common direct care tasks identified as being likely to be digitally enabled. Participants also discussed a range of quality of care-, staff-, and organization-related benefits and drawbacks in the use of technology. Finally, participants agreed that most of the researcher-proposed enablers and barriers were important, while suggesting additional enablers and barriers such as client preferences regarding technology use and poor data connectivity.

**Conclusions:**

This study provides insight into staff members’ views regarding the use of technology to deliver in-home aged care services. The results could help inform technology developers and in-home aged care providers, providing key information to guide technology implementation into care delivery. Further research is required to ensure that appropriate strategies are available to ensure successful implementation of technology into in-home aged care.

## Introduction

Globally, the older adult population is expected to continually grow over the next 25 years, with projections showing that the proportion of adults aged 65 years or older will increase to 16% in 2050 from around 10% in 2022 [1]. The World Health Organization has highlighted care at home as a key component in enabling older adults to age in a suitable environment and live a healthy and long life [2]. In Australia, this proportion is expected to be higher than the worldwide average, with 20% of people predicted to be aged 65 and older by 2031 [3]. This will drive a rapid rise in demand for in-home aged care services. In particular, the proportion of older-old Australians (aged 85 years and older), who may require additional services to remain safely at home, is expected to double from 2.1% in 2020 to up to 4.4% in 2066 [4], which may continue to drive demand for in-home aged care services.

Australian aged care services are generally delivered within a residential aged care facility, for individuals who have moved into aged care homes or the older person’s own home, where in-home aged care workers visit to provide care [5]. The number of Australian in-home aged care recipients, who receive a broad range of services, including domestic assistance, care coordination, personal care, social support, nursing, and allied health, to support their care needs, increased by 35% from 2017 to more than 1 million in 2023 [6]. Australia—like other countries—faces significant challenges related to the recruitment and retention of the aged care workforce, as well as funding of services [7]. Novel approaches to service delivery are required to meet the increasing demand for in-home aged care.

Technology has been suggested to have a vital role in improving the quality and transforming in-home aged care services to meet the rising needs of the older population [8]. A potential approach to use technology would be through digitally enabled care, which refers to using technology to augment new or existing care tasks within in-home aged care. A literature review has listed some common aspects of care in the in-home aged care sector that could be digitally enabled, such as falls prevention and detection, pain management, and care need assessments, using novel technologies including artificial intelligence, sensors, and interactive video games [9]. Research has shown that technology within in-home aged care can improve communication and care coordination, support the lone worker on the road and their geographically isolated clients, and enable social interactions for socially isolated clients [10]. For example, an Australian randomized controlled trial found that smart home technology improved social care–related quality of life in individuals receiving in-home aged care services [11]. With a significant proportion of Australian aged care recipients facing complex issues such as social isolation [12], and physical and mental health comorbidities [13,14], technology may serve as a crucial component of the solution to meet their needs.

Recent research has found that the majority of the current Australian in-home aged care workforce has sufficient digital readiness to use technology in the provision of care [15]. Despite this, and the potential benefits of technology within in-home aged care, the implementation and use of technology for aged care provision remain low [16]. Although the health and aged care sectors share many similarities, the aged care sector has lagged behind in the use of technological solutions [5,8]. There are suggestions that barriers such as ethical issues as well as individual, organizational, and system readiness impede the implementation of technology into aged care [9]. In addition, concerns have been raised about the potential impact of technology on the relational aspect of care, loneliness, inequities, and unintended consequences within in-home aged care services [10]. These concerns originated from apprehension surrounding the replacement of face-to-face contact with technology, ineffectiveness of technology to alleviate loneliness, unease around the accessibility of technology for older adults, and unforeseen consequences from lack of staff involvement when implementing technology [10].

A range of reviews have investigated the enablers and barriers of technology use in the health and aged care sectors. Enjoyment of technology, technology usability, personalization of the technology, familiarity with the specific technology, previous technology experience, support for the use of technology, and technology training acted as enablers for technology implementation into care from health or social care recipients’ perspective [17,18]. In contrast, technical issues, negative perceptions regarding technology use in care for older adults, limitations of the technology design, privacy concerns, technology usability, cost of technology, and lack of digital literacy were seen as barriers for home or health care recipients [17-19]. Although significant research has been conducted in the broader health, home care, and aged care sectors, there is a lack of evidence regarding the specific enablers and barriers of technology use in the in-home aged care setting from the perspective of the aged care workforce. Given the increasing role of the in-home aged care workers in both using technologies themselves and in supporting their clients to use technologies, this gap in knowledge requires attention. Specific research in the in-home care setting is required given its distinct characteristics, where providers deliver services to a cohort of older adults with unique care needs in their own homes, typically working on their own and without on-hand technical support.

At a time when several countries, including Australia, are developing strategies to increase the use of technologies to support health and aged care services, it is crucial to understand the potential of technology for direct care and the enablers and barriers of technology in the in-home aged care setting. This knowledge is critical for appropriate development of policy and implementation strategies and may also inform the development of future technologies to improve the quality of care provided to older people and enable them to remain safely at home for longer. Hence, this study aimed to understand in-home aged care staff members’ views of (1) the digital enablement potential of direct in-home care tasks, (2) benefits and drawbacks of technology use, and (3) enablers and barriers for technology use in Australian in-home aged care.

## Methods

### Design

An explanatory sequential mixed methods research design was selected to explore the digital enablement potential of direct care tasks and enablers and barriers for technology use within in-home aged care [20]. This study design was chosen to collect quantitative data first and then use qualitative methodology to explore the quantitative results in more depth [20].

### Cross-Sectional Survey

#### Research Objective

The aim of the survey was to understand staff members’ views on (1) the digital enablement potential of direct in-home care tasks, and (2) the importance of enablers and barriers for technology use within in-home aged care.

#### Sampling, Data Collection, and Measures

The sampling and recruitment procedure for the survey has been described in detail in a previous publication, which reported on a separate component of the survey [15]. In short, using convenience sampling, direct care in-home aged care workers from a large Australian in-home aged care provider were invited to participate in a staff survey between May and August 2023.

Specifically designed questions collected data on participants’ demographic characteristics, the digital enablement potential of direct in-home care tasks, and the importance of specific enablers and barriers for technology use within in-home aged care. For this study, direct care tasks were defined as tasks completed by in-home aged care staff that may require visiting a client’s home and support aged care recipients’ goals and care needs. For digital enablement potential, common direct care tasks for different roles were listed, with separate tasks listed for the key roles found in the Australian in-home aged care sector: managers or team leaders, Home Care Package coordinators or care managers, domestic assistants, care aides or therapy assistants, nurses, and allied health professionals. The list of common care tasks, which included items such as support with personal care, linen services, and clinical assessments, was developed by researchers through mapping of organizational service delivery documents used by a national in-home aged care provider, as well as Australian in-home aged care service guidelines [21-24]. Participants were asked to rate how likely it was that each of the listed direct care tasks could be digitally enabled (with existing or future technology) using a Likert scale from 1 = extremely unlikely, 2 = unlikely, 3 = likely, and 4 = extremely likely. Higher scores indicated a higher digital enablement potential.

A list of potential enablers and barriers was drafted by the research team through a comprehensive literature search focusing on potential enablers and barriers of technology use in home care settings and the research team’s clinician researchers’ lived experience working within in-home aged care. For example, “having staff involvement in how technology is implemented” and “cost to client” were listed as an enabler and barrier of staff technology use within in-home aged care, respectively. The full list of enablers and barriers is available in the “Results” section. Participants rated the importance of each enabler and barrier in technology use on a Likert scale from 0 = don’t know, 1 = not at all important, 2 = low importance, 3 = important, and 4 = very important. Higher scores indicated higher importance. In addition, open-ended questions were used to elicit responses from participants regarding additional enablers and barriers related to technology use within in-home aged care.

#### Data Analysis

Descriptive statistics were generated using frequencies and percentages for categorical variables and means and SDs for continuous variables in Stata (version 18.0 BE; StataCorp). The digital enablement potential of tasks was ranked from highest to lowest for each role based on the mean score of each task. Similarly, the importance of enablers and barriers for technology use was ranked from highest to lowest using the mean score. Participants who rated zero (don’t know) for the enablers and barriers were removed from analysis for the specific enabler or barrier. Open-ended questions were coded inductively using applied thematic analysis in NVivo (version 12.0; Lumivero) [25]. Applied thematic analysis is a pragmatic, inductive qualitative data analysis technique [25]. The 5 elements of applied thematic analysis (text segmentation, codebook creation, structural coding, content coding, and theme development) were used during analysis [25].

### Semistructured Staff Interviews

#### Research Objective

The staff interviews aimed to describe (1) the digital enablement potential of direct in-home care tasks, (2) benefits and drawbacks of technology use, and (3) enablers and barriers for technology use within in-home aged care.

#### Sampling

A purposive sampling approach was used for the semistructured interviews. Current in-home aged care staff from a single provider organization working in South Australia, Victoria, or Western Australia were eligible to participate and recruited in 2 ways. First, individuals who completed the staff survey and opted in for further research were invited to participate in a semistructured interview. Second, managers were contacted to nominate in-home aged care workers from different roles to be invited to participate in the interviews. This approach ensured that participants from a diverse range of roles and locations were recruited to participate in the study. In both cases, invitations were sent to potential participants via email, with the participant information and consent forms. Interested staff were asked to respond to the email, provide a signed consent form, and arrange a suitable time for the interview.

#### Data Collection

Web-based semistructured interviews were conducted using Microsoft Teams by SW and BK. The interviews included questions related to the digital enablement potential of direct care tasks, benefits and drawbacks of technology use, and enablers and barriers for technology use within in-home aged care. The interview schedule is available in Multimedia Appendix 1. As the research team began to conduct interviews during the survey period, the indicative aggregate survey results were used as interview materials to elicit feedback and discussion from the interviewees. Aggregate responses to open-ended questions in the survey regarding additional enablers and barriers were also presented for discussion. These interview materials are also available in Multimedia Appendix 1. The interviews were transcribed verbatim using Microsoft Teams.

#### Data Analysis

Data were entered into NVivo and analyzed using applied thematic analysis [25]. Analysis was conducted by BK, with MD conducting double coding of 20% of the transcripts to ensure rigor of coding. The research team familiarized themselves with the data by reading the transcripts. The codebook was initially constructed based on the answers to the survey questions and then further developed iteratively to develop and analyze the relationships between codes and themes.

### Positionality Statement

BK is an in-home aged care physiotherapist with qualitative research training. MD and TD are researchers embedded within an in-home aged care provider, with TD having a clinical psychology background. SW, FB, and KH are researchers at a national aging research institute, with SW and FB having clinical experience as physiotherapists in health care and aged care settings. KF has experience working as an in-home aged care pharmacist and developing innovative models of care. TW and HJ are consumers with lived experience of in-home aged care.

### Ethical Considerations

Ethics approval was granted by the Silverchain Low Risk Ethics Committee (RG-054). Informed consent was obtained from participants prior to study participation through the provision of a participant information and consent form. All collected data were deidentified during data analysis and project reporting. No financial compensation was given to the participants of the survey. Financial compensation was provided to the operational team to backfill staff participation in the interviews.

## Results

### Descriptive Statistics

For the survey, 226 participants consented and completed questions relevant to this study. Eighteen participants consented to participate in the interviews. Descriptive statistics of both groups of participants are shown in Table 1. In the survey, 69.5% (157/226) of participants were aged 45 years or older, and 89.8% (203/226) were female. The majority worked in a part-time or casual position (144/226, 63.7%), with a broad range of different roles reported. Across the survey and interviews, most participants worked in metropolitan areas (162/226, 71.7% and 11/18, 61.1%, respectively), with approximately half of them having 5 or fewer years of experience working within in-home aged care (110/226, 48.7% and 10/18, 55.6%, respectively).

**Table 1.** Detailed sociodemographic characteristics (frequency, %).

Characteristics	Staff survey (N=226), n (%)	Staff interviews (N=18), n (%)
Total participants	226 (100)	18 (100)
Age group (years)		N/A^a^
18‐24	6 (2.7)	
25‐34	21 (9.3)	
35‐44	42 (18.6)	
45‐54	71 (31.4)	
55‐64	75 (33.2)	
65+	11 (4.9)	
Gender		N/A
Male	21 (9.3)	
Female	203 (89.8)	
Nonbinary/third gender	1 (0.4)	
Prefer not to say	1 (0.4)	
Employment type		N/A
Full-time	82 (36.3)	
Part-time	123 (54.4)	
Casual	21 (9.3)	
Location of work		
Metropolitan	162 (71.7)	11 (61.1)
Regional, rural, and remote^b^	N/A	7 (38.9)
Regional	29 (12.8)	N/A
Rural	25 (11.1)	N/A
Remote	10 (4.4)	N/A
Education		N/A
Some secondary schooling	10 (4.4)	
Senior secondary schooling (completed high school or equivalent)	48 (21.2)	
Trade/technical/vocational training or diploma	90 (39.8)	
Bachelor’s degree (including Honours)	49 (21.7)	
Postgraduate degree (eg, master’s degree, PhD, or professional degree)	16 (7.1)	
Missing	13 (5.8)	
Length of time working within in-home aged care (years)		
0‐1	35 (15.5)	3 (16.7)
2‐5	75 (33.2)	7 (38.9)
6‐10	32 (14.2)	0 (0)
11‐15	32 (14.2)	1 (5.6)
16‐20	28 (12.4)	4 (22.2)
21‐25	9 (4.0)	1 (5.6)
25+	15 (6.6)	2 (11.1)
Main role		
Allied health professional	28 (12.4)	3 (16.7)
Care aides/therapy assistants	95 (42.0)	3 (16.7)
Enrolled nurses/registered nurses	5 (2.2)	1 (5.6)
Domestic assistants	51 (22.6)	1 (5.6)
Home care package coordinators/care managers	28 (12.4)	6 (33.3)
Team leader/manager	19 (8.4)	4 (22.2)

a N/A: not available.

b Regional, rural, and remote were categorized together for the purposes of the interviews.

### Digital Enablement Potential of Direct In-Home Aged Care Tasks

The mean digital enablement potential of each direct care task is reported in Table 2, with illustrative quotes regarding representative tasks from the interviews presented alongside the quantitative results. Overall, 56% (14/25) of the listed direct care tasks were rated a mean of 3 or above, indicating that participants felt that many direct care tasks could be digitally enabled within in-home aged care. This was reinforced by the interviews, with most interview participants reporting that some direct care tasks could be digitally enabled, but this varied across roles. Notably, participants who worked as a care aide or a therapy assistant ranked all their listed tasks to a mean of below 3 (ranging between 2.22 and 2.82), and participants who worked as a domestic assistant ranked their listed tasks below or just slightly above a mean of 3 (ranging between 2.35 and 3.04), indicating these subgroups of in-home aged care staff felt that their tasks were less likely to be digitally enabled. For example, a participant working as a domestic assistant reported:

*I see my work as hands on.*

[Interview participant 17, Care aide/therapy assistant]

Survey participants in clinical or care coordination roles, such as allied health professionals and care coordinators, rated communication-related tasks in their roles relatively more likely to be digitally enabled than noncommunication tasks, with communication-related tasks with staff or external health care providers rated as the top or second most likely task to be digitally enabled. While managers and team leaders rated communication with team members as the task most likely to be digitally enabled, they also rated client communication as the least likely task to be digitally enabled. In contrast, interview participants reported that communication with clients is currently digitally enabled, using common technology such as text messages or emails.

*I think a huge one for me [as] the [home care] package coordinator. There’s actually emailing . and that’s with clients*.

[Interview participant 07, Home care package coordinator/care manager]

In addition, in the survey, allied health professionals and nurses rated clinical assessment, which was ranked first or second, more likely to be digitally enabled than clinical treatment, which was ranked last for both professions. However, interview participants voiced a range of different opinions regarding the digital enablement potential of clinical treatments:

*Nursing treatments. Obviously, that’s...face to face*.

[Interview participant 09, Home care package coordinator/care manager]

*In terms of social work...we could deliver a fair amount of education and treatment digitally*.

[Interview participant 02, Allied health professional]

While the majority of tasks conducted by domestic assistants were considered unlikely to be digitally enabled, an exception was unaccompanied shopping services, which could be digitally enabled. For example, a participant (occupational therapist) suggested that unaccompanied shopping services could be digitally enabled to empower in-home aged care recipients.

*The other programs have been helping people with shopping online, so people who are unable to leave home...but with support they can...I have heard good feedback about that...You know the two people are sitting there in front of the computer doing it together, but I think that’s that is a good example of an enabling...task [because] you’re helping that person to choose their own shopping...so I think it’s important*.

[Interview participant 11, Team leader/manager]

While many direct care tasks were seen as having potential to be digitally enabled, some participants expressed concern that the quality of the service delivered may be decreased or the service may not achieve its intended purpose:

*[Virtual occupational therapy] assessments, while they can be done and I have done them, [they’re] definitely not as easy as in person and you don’t get the same depth and richness of information*.

[Interview participant 11, Team leader/manager]

*It’s interesting that...by thinking that just by seeing a face online [during social support services] is going to solve the problem. But as you say, maybe it’s more the actual going to the cafe and seeing the world and having that coffee as much as that person that they’re with, but it’s a combination of everything rather than just the face*.

[Interview participant 08, Home care package coordinator/care manager]

**Table 2.** Likelihood of digitally enabling direct care task^a^ (n=226; ranked from highest to lowest likelihood in each role).

Direct care task	Score, mean (SD)	Representative quotes
Managers/team leads (n=19)		Staff management: “It’s not brilliant...just to have one team on a video call and then the others in the room. It would be better to have everyone in the room, but...it’s a way that...we can still interact, so it’s better than nothing*”* [Interview participant 09, Home care package coordinator/care manager].
Communicating with team members	3.21 (0.63)	
Staff management	3.16 (0.60)	
Onboarding/training team members	3.16 (0.77)	
Client communication	2.79 (0.63)	
Home care package coordinators/care managers (n=28)		Care coordination: “Technology is really beneficial in that being able to do things like email or...Teams meetings...you can...fit things into a busy schedule a lot easier. And a lot of my clients have kids who are still working but still really [want to] provide input and be collaborative on services. But you know, when I’m working full time and they’re working full time, it’s hard to kind of find for them to take time off to do whatever, so it can really facilitate carer and family involvement in services as well” [Interview participant 02, Allied health professional].
Liaising with other health professionals	3.50 (0.51)	
Staff management	3.21 (0.79)	
Care coordination	3.11 (0.83)	
Client assessment	3.04 (0.96)	
Domestic assistants (n=51)		General house cleaning: “You need to physically clean a house” [Interview participant 17, Care aide/therapy assistant].
Unaccompanied shopping (delivered to home)	3.04 (0.60)	
Linen services	2.45 (0.73)	
General house cleaning	2.35 (0.80)	
Care aides/therapy assistants (n=95)		Assistance with self-administration of medications: “Medication administration that is in a dispenser and the dispenser is...electronic and it dispenses the tablets at the right time and tells them when to get up. [The client will] know when to take them” [Interview participant 01, Home care package coordinator/care manager].
Assistance with self-administration of medications	2.82 (0.67)	
Support with mobilizing (eg, getting in and out of bed/moving around the house)	2.72 (0.75)	
Support with transport	2.68 (0.73)	
Assistance with allied health therapy	2.67 (0.57)	
Social support	2.54 (0.73)	
Support with personal care (eg, toileting, bathing, dressing, and grooming)	2.22 (0.84)	
Enrolled nurses/registered nurses (n=5**)**		Nursing assessment: “We have attempted some remote clinical assessments via [Microsoft] Teams which are OK, but connection drops out sometimes” [Interview participant 09, Home care package coordinator/care manager].
Nursing assessment	3.60 (0.55)	
Liaising with other health professionals	3.40 (0.55)	
Client education	3.20 (0.84)	
Nursing treatment	2.60 (1.14)	
Allied health professionals (n=28)		Allied health assessment: “My allied health assessments, all of my assessments are done on my laptop. I don’t feel like I could go into an assessment, write on paper and then come back out and do that onto my laptop. I feel like that would be such a waste of time” [Interview participant 14, Allied health professional].
Liaising with other health professionals	3.50 (0.58)	
Allied health assessment	3.43 (0.63)	
Client education	3.25 (0.75)	
Allied health treatment	2.89 (0.79)	

a Extremely unlikely = 1, unlikely = 2, likely = 3, and extremely likely = 4.

### Benefits and Drawbacks of Technology Within In-Home Aged Care

The benefits and drawbacks of technology within in-home aged care were explored in semistructured interviews, with findings summarized in Table 3. Findings were categorized into benefits and drawbacks relating to quality of care, staff, and organization.

**Table 3.** Benefits and drawbacks of technology within in-home aged care and representative quotes from semistructured interviews.

Benefits and drawbacks	Representative quotes
Quality of care–related benefits	
Improving availability of care	“Considering how big and remote [Western Australia] is...probably be an advantage just because for them to get to sort of someone else to see it might be next to impossible” [Interview participant 12, Enrolled nurse/registered nurse].
Increasing data available to inform care	“They can actually track to see whether they’re actually having [the medication] or not” [Interview participant 16, Domestic assistant].
Meeting client preferences regarding technology-supported care	“Depending on the client and what they are wanting because it is [all] about delivering best care to each and every client and we want to make sure that they’re happy and living the life that they want to live, so if it could change, maybe [having technology in their care] would be the way forward” [Interview participant 10, Team leader/manager].
Reducing cost of care for clients	“I think it’s very valuable for administrative purposes for doing...reviews to help cut down on time and therefore cost to client” [Interview participant 11, Team leader/manager].
Improving flexibility of scheduling of care	“I guess we should always give the clients the option of whether or they want us like for example, if we’re doing...a virtual...session rather than face to face...There will be some clients who, especially if they’re time poor and...they might not get up early in the morning they want you to...see them at like 3:30 in the afternoon for example...there might be a bit more flexibility around times if we don’t have to then get back to the office and finish work” [Interview participant 05, Allied health professional].
Empowering clients’ independence and function	“If we could show them, I think the benefits of some of these things like the banking and things like that and that they can do online shopping...I think the [technology] would make them also feel a bit more independent as well, because I think they have to rely on technology a lot and they don’t understand it” [Interview participant 01, Home care package coordinator/care manager].
Enabling clients and carers to be more involved in their care	“I have clients that don’t speak English and the family member might [want to] be there to help out...We also have access to interpreters, but often they prefer to have a family member there. The family member might be they could be Interstate...they might be on the other side of town. [You] could even do it like as a 3-way conversation [using technology]” [Interview participant 05, Allied health professional].
Improving client safety and security	“Wearables have been amazing for our clients...We started off people having personal alarms around their neck that they had to press if they’ve had a fall. Now we’ve got wearables that will alert family members when clients have had a fall and let them know where they are, so I think technology is assisting with giving us fantastic care for our client group*”* [Interview participant 04, Home care package coordinator/care manager].
Staff and organization–related benefits	
Helping the organization continually improve	“You know it’s going to be a stepping stone for what we’re evolving into as a company, as an organization” [Interview participant 10, Team leader/manager].
Improving accessibility to up-to-date information and data	“The software that we use, you live by it in a way because it’s got your schedule, it’s got every detail that you need to know” [Interview participant 10, Team leader/manager].
Improving flexibility of staff rostering	“Because we’ve got the roster on our phones, which I find really much, much better...in the sense that we can kind of like change things a little bit without...being ridiculous about it but adjust things so that everything fits nicely on for the day” [Interview participant 18, Care aide/therapy assistant].
Improving staff efficiency	“It’s really good to be able to do more regular follow up because...you don’t have to be driving everywhere. You’re not spread as thin. And again, good for staff shortages” [Interview participant 11, Team leader/manager].
Meeting the needs of on-the-road staff	“The laptop really does help, because we need to be portable, we need to be mobile. You know, our laptops are connected to the Internet, which is really helpful to have a SIM card built in” [Interview participant 11, Team leader/manager].
Quality of care–related drawbacks	
Impact rapport building due to having technology	“I take my laptop in and sometimes I feel that it hinders building that relationship with a client because I’ve got a screen in front of me. I work sometimes from both my laptop and my phone at the same time, so I feel like they are not giving me their full story because...I look like I’m playing on my phone or playing on my laptop rather than building that eye contact, building that relationship with the client” [Interview participant 14, Allied health professional].
Impact on client privacy with use of technology for safety	“I have seen a couple of client situations where family members have put cameras around that they can’t be in the same house. They’ve put a lot of cameras with motion sensors and those sort of things for clients with dementia, and I find that quite intrusive as a way of caring for somebody. So that use of technology I find a bit jarring” [Interview participant 04, Home care package coordinator/care manager].
Reducing human interaction during care	“We can’t have robots going in and seeing the clients for us, we still need...that might come eventually one day, but we still need the face-to-face human interaction...We need to keep that face-to-face. Definitely” [Interview participant 16, Domestic assistant].
Reducing time for care	“Sometimes it’s the time spent on technology that might not be good...You lose time to spend on care” [Interview participant 17, Care aide/therapy assistant].
Technology issues may negatively impact quality of care	“[Electronic clinical records] can be very glitchy. It’s already happened to me this morning where it had some sort of fatal error and booted me out. I had to log back in and start from scratch again and that all takes time. Uh, it can be very slow to download documents...and things like that. If you need to access documents like medication orders...that have been uploaded [but] haven’t seen yet that are on [the electronic clinical records], that can be very slow to access” [Interview participant 12, Enrolled nurse/registered nurse].
Increasing clients’ functional decline and care needs	“I think that implementing too much technology into home care. I think I would have to disagree with it...getting their...services like...social support...and replacing it with technology, I think that could be really a part of a client’s decline, so they could start getting depressed or feel socially isolated” [Interview participant 08, Home care package coordinator/care manager].
Staff and organization–related drawbacks	
Increased physical burden due to technology use	“You don’t [want to] be [staring] at a screen all day. Mainly so it does become quite hard*” *[Interview participant 08, Home care package coordinator/care manager].

#### Quality of Care–Related Benefits and Drawbacks

Several quality of care–related benefits were discussed by interview participants, including benefits for care delivery, as well as direct benefits for clients. Care delivery benefits included improvements to the availability of care, better access to data to inform care, and greater flexibility of scheduling of care (Table 3). In addition, participants suggested that technology use within in-home aged care can empower clients’ independence and function, enabling them to be more involved in their care and allowing them to remain connected to the world.

*I think that’s a great idea because clients are always wanting to keep in the new age. They don’t want to feel like they’re going back to the old ways, as they say. So, helping them or supporting them to be able to access [technology] and be able to know what’s happening in the world and then I think it’s a really good benefit for them*.

[Interview participant 10, Team leader/manager]

In contrast, participants reported that there were several drawbacks that could arise from technology use within in-home aged care, including the potential for technical issues with technology to negatively affect the quality of care:

*What we do is often we’ll obviously restart, try to re-establish a connection or...abandon the video call and then go through to the phone call. When we do abandon the video call, obviously we can’t see the client, so we can’t do necessary skin checks and...more visual type assessments that we can do via video call...That does impact our assessment*.

[Interview participant 09, Home care package coordinator/care manager]

Many participants discussed concerns that technology use could result in less time to spend delivering in-person care, impacting on their ability to build a meaningful relationship with the client, and reducing interpersonal interaction during direct care delivery. This participant felt that many older people rely on aged care providers for regular human interaction:

*The thing that you just miss is that real human connection of being in a room with somebody. And for a lot of our older adults, [the] only connection with [a] person on a regular basis is when someone comes in to help him with their shower or do their shopping or cook their meal. And I think if you switch now while you could never help someone with their shower virtually, there are lots of things you could cut out. And I just feel the person would lose that. That’s where the quality goes. It’s that human connection*.

[Interview participant 11, Team leader/manager]

#### Staff and Organization–Related Benefits and Drawbacks

Similarly, participants suggested that technology could bring multiple benefits for staff and in-home aged care organizations (Table 3). Several participants proposed that technology use can improve the accessibility of up-to-date information and data, including access to care-relevant information, such as information on client preferences regarding their care.

*If I was to go in...and do an assessment, and then I would put on [the electronic medical record] alerts, this is a danger I could put in a care plan. This is the way that we should probably [be] providing the care, so the next provider would have all of that information. I think that would enhance the care [provided to the client]*.

[Interview participant 14, Allied health professional]

*There can also be alerts on what a client’s preferences are, so you can already...be equipped with the tools to go in and provide the best care possible with...access to that information*.

[Interview participant 14, Allied health professional]

Furthermore, many participants discussed the improvements to staff efficiency that could be provided through technology use, such as reduction of time spent on travel and administrative tasks.

*I think it’s very valuable for administrative purposes for doing...reviews to help cut down on time*.

[Interview participant 11, Team leader/manager]

*It’s [going to] make us more efficient...Let’s say the average day...we’re expected to do X hours face to face [client] time...If some of that time [we] could still [be] in the office doing an assessment or a review via this kind of [technology] arrangement, then...we will be able to fit in more clients because we’re not [wasting] time traveling*.

[Interview participant 05, Allied health professional]

Conversely, interview participants proposed only 1 staff-related drawback of technology use within in-home aged care. Participants reported that technology use can result in increased physical burden related to sitting at a computer or using a device screen for extended periods:

*It is unpleasant having to do so much with mobile. [My] neck gets very sore looking down all the [time]*.

[Survey participant 11]

### Enablers and Barriers for Technology Use Within In-Home Aged Care

Enablers and barriers for technology use were explored in both the survey and the interviews. The quantitative findings from the survey are reported in Tables 4-6. Illustrative quotes from survey and interview participants regarding the enablers and barriers are reported in Table 7. Overall, the survey participants rated all the listed enablers (ranging between 3.12 and 3.81) and the majority of listed barriers (ranging between 2.92 and 3.74) as having an important impact on their use of technology within in-home aged care. In the interviews, participants also mostly agreed with enablers and barriers listed in the survey.

**Table 4.** Enablers of staff using technology (n=199, missing=27; ranked from highest to lowest importance)^a^.

Enablers	Score, mean (SD)	
Having someone to go to if or when the technology does not work	3.81 (0.41)	
Having technology that is reliable	3.79 (0.43)	
Having technology that is suitable and appropriate	3.69 (0.49)	
Having good organizational and managerial support	3.62 (0.57)	
Having staff involvement in how technology is implemented	3.57 (0.55)	
Having enough time to get familiar with the technology	3.56 (0.58)	
Having education and training on how to use technology for care tasks	3.51 (0.62)	
Your confidence using technology for care tasks	3.34 (0.66)	
Your previous experience in using technology for care tasks	3.12 (0.64)	

a Don’t know = 0, not at all important = 1, low importance = 2, important = 3, and very important = 4.

**Table 5.** Barriers of staff using technology (staff-related factors) (n=199, missing = 27; ranked from highest to lowest importance)^a^.

Barriers	Score, mean (SD)	
Having unreliable technology	3.74 (0.59)	
Having limited or no support when technology does not work	3.70 (0.60)	
Having inappropriate or unsuitable technology	3.69 (0.64)	
Having limited or no staff involvement in how technology is implemented	3.48 (0.69)	
Having limited organizational and managerial support	3.46 (0.72)	
Having limited time to get familiar with the technology	3.46 (0.75)	
Limited education and training on how to use technology for care tasks	3.38 (0.76)	
Cost to client	3.17 (0.90)	
Concern that introducing technology will increase to workload	3.08 (0.79)	
Your confidence using technology for care tasks	3.05 (0.90)	
Client ability, familiarity, and confidence using technology	3.04 (0.83)	
Client health status	2.96 (0.87)	
Your previous experience in using technology for care tasks	2.92 (0.84)	

a Don’t know = 0, not at all important = 1, low importance = 2, important = 3, and very important = 4.

**Table 6.** Barriers of staff using technology (client-related factors) (n=199, missing = 27; ranked from highest to lowest importance)^a^.

Barriers	Score, mean (SD)	
Cost to client	3.27 (0.88)	
Client ability, familiarity, and confidence using technology	3.23 (0.88)	
Client health status	3.13 (0.84)	

a Don’t know = 0, not at all important = 1, low importance = 2, important = 3, and very important = 4.

**Table 7.** Enablers and barriers of staff using technology and representative quotes from semistructured interviews.

Enablers and barriers	Representative quotes
Having someone to go to if or when the technology does not work/Having limited or no support when technology does not work	“We have a really good IT department, so if it is...my computer is not turning on or my phones [is] not doing something, I will always send them to IT when it is IT specific” [Interview participant 11, Team leader/manager].
Having technology that is reliable/Having unreliable technology	“When you get to a client and it’s not loaded in properly and you might have to restart your phone. Then you’ve just got to wait for it to have to do that, and sometimes it might cut into the client’s time, or you’re stood there waiting because you’ve [got to] wait for your phone to log in, so you can log in and start that client’s visit” [Interview participant 10, Team leader/manager].
Having technology that is suitable and appropriate/Having inappropriate or unsuitable technology	“The program we have is pretty comprehensive. It’s got all our schedule, and it’s got all the maps and everything...I plug it into my car with Android Auto, but hit the map thing and it directs me to my next client, so it makes it easier to [find and] not having to go and hunt up addresses and do all that thing and type anything in to actually do that. That makes it really simple” [Interview participant 12, Enrolled nurse/registered nurse].
Having good organizational and managerial support/Having limited organizational and managerial support	“We do have quite good managerial support in terms of the technology” (Interview participant 02, Allied health professional)./“We did do a trial with the IT [department and they] were quite supportive” [Interview participant 11, Team leader/manager].
Having staff involvement in how technology is implemented/Having limited or no staff involvement in how technology is implemented	“The frontline staff are often the ones that are left out or it’s only trialed with a small...area of the frontline staff. And I don’t think that gives a broad understanding of the bigger picture” [Interview participant 13, Team leader/manager].
Having enough time to get familiar with the technology/Having limited time to get familiar with the technology	“When you’re starting the job...getting used to the program is a big part. I’ve been training the staff member for a couple of weeks now and it even when I...started it probably took me a good couple of months to find all the little different bits and pieces and shortcuts and things like that*”* [Interview participant 12, Enrolled nurse/registered nurse].
Having education and training on how to use technology for care tasks/Limited education and training on how to use technology for care tasks	“Yeah, I think we definitely need to look at doing it better. Because I think with a lot of these programs, you know you spend so much time on it...building them and implementing them. And then it’s only a short window of training to make sure everybody’s on board and then off you go. You’ll be right. And like I said, and then it’s the staff on the ground that are left picking up the pieces like, yeah, I’ll walk you through that. So that’s probably a big gap is training on digital for sure” [Interview participant 07, Home care package coordinator/care manager].
Your confidence using technology for care tasks	“I think it’s what we just spoke about like...making sure they’ve got competence and the domestic [worker] actually go out there and basically given the tools to start using that technology and not be scared to use [the] technology*”* [Interview participant 16, Domestic assistant].
Your previous experience in using technology for care tasks	“Some of them don’t even have a smartphone that they use in their own life, so having to explain how to use a mobile phone is quite tricky as part of an onboarding process” [Interview participant 13, Team leader/manager].
Cost to client	“Cost to client...Yeah, there’s not a lot. Well, I wouldn’t say that there’s a huge cost to client because you know not in our setting because they would just have a normal clinical assessment. They’d be billed for that. They wouldn’t be [billed] for the technology aspect” [Interview participant 09, Home care package coordinator/care manager].
Concern that introducing technology will increase to workload	“We’ve attempted some remote clinical assessments via teams which are OK, but connection drops out sometimes. You know, if clients have dementia or they’ve got cognitive issues, they obviously can’t control the technology themselves, so we have to rely on external or other people to assist us with that and that becomes an issue. That’s sort of put...a stop to us doing it because it’s taking more time for...staff to sit in the home with the clients while we do our work” [Interview participant 09, Home care package coordinator/care manager].
Client ability, familiarity, and confidence using technology	“Familiarity and confidence with using [technology]. That’s a big one. I think people get scared of technology and especially the elderly*”* [Interview participant 09, Home care package coordinator/care manager].
Client health status	“If clients have dementia or they’ve got cognitive issues, they obviously can’t control the technology themselves” [Interview participant 09, Home care package coordinator/care manager].
Additional enablers	
Client preference of technology	“People are asking for it...And especially our Parkinson’s clients that are looking at, ‘This is my future. I’m [going to] need a computer to be able to get my meals into and I don’t know how to do that and to be able to communicate with my family and to be able to...Our speech [pathologist] at the moment, go out and do a little bit with clients’” [Interview participant 06, Home care package coordinator/care manager].
Paced introduction of technology into care	“I just think that it’s definitely the way [of] the future and as long as it’s introduced slowly into people’s lives...I think as long as it’s introduced slowly, people are much more open to it. And the more you kind of introduce, the more people will accept it” [Interview participant 14, Allied health professional].
Additional barriers	
Client preference of technology	“Some of them don’t like it and send it back. It’s not what they wanted” [Interview participant 01, Home care package coordinator/care manager].
Lack of technology infrastructure for clients	“They either don’t have an Internet connection, they don’t have smartphones, or they don’t feel comfortable using it to do phone calls like that or telehealth. Most who do telehealth and even during COVID, they were supported by a family member...I don’t [want to] say none, but the large majority just don’t have the technology yet preexisting in their home” [Interview participant 11, Team leader/manager].
Lack of client trust of technology	“The most part of it is they don’t trust technology, and that’s what they say. Because with all the scams that goes around, they just don’t have that belief that it’s a safe way for communication” [Interview participant 10, Team leader/manager].
Digital literacy of staff	“I would say to understand the differences between different software and how to manage them, because it is quite common for staff members not to be very tech savvy with them*”* [Interview participant 10, Team leader/manager].
Staff preference regarding the technology use in care	“It should be something that’s not mandatory...I believe that it should be something that individual clinicians should be able to do if they are confident to do so. If clinicians want to do that, but aren’t confident, then there should be training offered because it’s going to make us more efficient*”* [Interview participant 05, Allied health professional].
Complexities related to the aged care system	“The only issue I would see with that is when we get agency staffing or like agency nurses, we have a lot of brokerage staff. For example, if we don’t have enough cleaners, we might go to another agency...so they don’t wouldn’t be privy to being able to see what’s on [electronic care plans]” [Interview participant 06, Home care package coordinator/care manager].
Cost to the organization	“Cost is huge, and that’s always going to be [a] huge cost to the organization and cost to client because you know it’s always going to be the biggest barrier. I think everybody wants bang for their buck and it’s not always [going to] be at the top of the list” [Interview participant 04, Home care package coordinator/care manager].
Having poor data connectivity	“This is...definitely a limiting thing in some areas, and downloading different stuff can take quite a long time or just doesn’t work” [Interview participant 12, Enrolled nurse/registered nurse].

In the survey, the 3 highest ranked enablers for staff use of technology within in-home aged care were having someone to go to if or when the technology does not work (mean 3.81, SD 0.41), having technology that is reliable (mean 3.79, SD 0.43), and having technology that is suitable and appropriate (mean 3.69, SD 0.49) (Table 4). Similarly, albeit in a different order, the 3 highest ranked barriers for staff technology use were having unreliable technology (mean 3.74, SD 0.59), having limited or no support when technology does not work (mean 3.70, SD 0.60), and having inappropriate or unsuitable technology (mean 3.69, SD 0.64) (Table 5). Other factors, such as organizational support, staff involvement in the implementation of the technology, and opportunities for training and familiarization with technology, were also strongly endorsed as enablers (or barriers when not present).

Participants highlighted the importance of having support in how to use technology. Some participants also suggested that unofficial peer support from colleagues facilitated technology use, as well as more formal organizational support, for example, from their manager or IT department.

*Having someone to go to when technology doesn’t work is vitally [important]. I ring IT on a very regular basis*.

[Interview participant 12, Enrolled nurse/registered nurse]

*And I was constantly calling other team members...Just “How do you do this?,” “How do you do that?” and having within my own team someone that I could call to get that advice was very helpful*.

[Interview participant 12, Enrolled nurse/registered nurse]

The reliability of technology was highlighted by participants as important, with participants reporting additional time and work are required when the technology is unreliable.

*A software, say like one of our scheduling software. Sometimes that does [crash] on you, and sometimes you have to load out of it and load back in, so it would be nice to have something that runs very smoothly and is reliable*.

[Interview participant 10, Team leader/manager]

Different aspects of the suitability and appropriateness of technology were discussed by participants, such as the design of technology and the intersection of technology with the features of in-home aged care. The participants highlighted the importance of the suitability of technology design for the on-the-road nature of in-home aged care and the direct care tasks delivered to aged care clients. For example, one participant talked about the limitations of a smartphone for some tasks, whereas another participant discussed the suitability of a software for in-home aged care:

*The phones are ridiculously hard to use if we’re trying to seek information...because it’s so little and small to try and either type on if you’re out in the field or try to navigate through the system to try and get the information that you need. That’s been a hindrance*.

[Interview participant 06, Home care package coordinator/care manager]

*I think it’s good for being on the road because it gives you all the information that you need*.

[Interview participant 10, Team leader/manager]

Participants also raised additional enablers and barriers to technology use by staff within in-home aged care. Client preferences regarding the use of technology emerged as both an enabler and a barrier. Participants reported that aged care clients have differing preferences for technology, with some clients requesting more technology use and others requesting technology be removed from their home. Other barriers to technology use discussed included lack of technology infrastructure for clients, lack of client trust, and staff digital literacy and preferences. Poor data connectivity was highlighted as a barrier by multiple participants:

*Depending on where you are in Perth, unfortunately there are some black spots around and we have no coverage...This is...definitely a limiting thing in some areas, and downloading different stuff can take quite a long time or just doesn’t work...There are some spots where we just have no coverage and there’s nothing you can do in that situation*.

[Interview participant 12, Enrolled nurse/registered nurse]

*We have a lot of issues with coverage in certain areas*.

[Interview participant 13, Team leader/manager]

## Discussion

### Principal Results

In this study, we used a mixed methods approach to investigate Australian in-home aged care workers’ views on the digital enablement potential of direct care tasks, benefits and drawbacks of technology use, and the enablers and barriers to technology use within in-home aged care. Overall, participants felt that many direct care tasks within in-home aged care could be digitally enabled, with more than half of the common direct care tasks completed by staff identified as being likely to be digitally enabled. This is in line with research findings from Ireland and Spain, which show that health and social care professionals appear positive and hopeful regarding the role and use of technology in care delivery within health and social care [26].

However, our study found that staff members’ views on the digital enablement potential of their tasks differed by professions, with care aides, therapy assistants, and domestic assistants rating their tasks less likely to be digitally enabled. This is similar to research conducted in the Finnish setting, which suggested that care workers’ views on the digital enablement potential may be partially due to staff in these roles viewing their work to be more physical and hands-on in nature [10]. This is an interesting finding as new technology, such as robot vacuums (which are available for Australian in-home aged care clients to access as part of their services) [27], is becoming increasingly common and could complement direct care tasks, such as general house cleaning. Previous research has found that Australian in-home aged care workers’ role was associated with their digital readiness, with staff with less clinical responsibilities having higher proportions of individuals with lower digital readiness [15]. This may have negatively influenced participants’ views on the digital enablement potential of their direct care tasks. Therefore, some in-home aged care workers may require additional support to improve their digital readiness and, in turn, change their views on digitally enabling direct care tasks. This study showed that staff in clinical or care coordination roles felt that communication tasks with colleagues or external health providers had a high digital enablement potential. This concurs with the existing literature that technology in home care services could enhance communication and coordination and free up time for more care provision [10,26].

Despite the overall positive views from staff regarding the digital enablement potential of direct care tasks, concerns were raised regarding the potential impact of digital enablement on the quality of care. In the interviews, staff discussed multiple quality of care-related benefits from the use of technology within in-home aged care, such as improved care availability, increased access to care information, client enablement, and improved client safety and security. On the other hand, staff were concerned that digital enablement could have drawbacks, including reduced human interactions during care, impact on client privacy, and the negative impact of technology issues on care quality. Similar to our results, research from Finland and Ireland showed that there were concerns from health and social care providers that the increasing use of technology would lead to the loss of the “human touch” and reduce human interactions during care [10].

Interestingly, government strategies supporting technology in aged care have suggested that technology would be able to reduce the time spent on noncare activities such as administration to free up time for direct care [28]. However, the mismatch between the government and the workers’ views may stem from the wide range of technology that could be implemented within in-home aged care, which may have different impacts on human interactions during care. The potential loss of “human touch” that might occur with technology would need to be carefully considered to avoid negative impacts on the quality of in-home aged care. Participants also discussed benefits and drawbacks of digital enablement related to staff and the organization. One such benefit of technology is the improved accessibility of up-to-date information. This is consistent with previous research showing that digitalization in health and social care services could improve the care-related information availability for staff regarding care recipients [26].

In order to realize the potential benefits of technology, factors related to technology use need to be considered during the implementation of technology into in-home aged care. The majority of the study cohort agreed with the importance of most of the enablers and barriers generated by the research team and suggested additional enablers and barriers from their experience of working in Australian in-home aged care. The most important factor for staff technology use was the availability of support when technical issues occur. The different types of support that were important for staff use of technology were “official” support provided by the internal information technology department and “unofficial” support provided by their colleagues. As shown in an Australian telehealth trial, clinicians require support for a wide range of technical tasks, such as software and hardware issues, assistance with technology setup, and device management [29]. It is critical for technical support to be readily available for staff as and when required. Another barrier raised by multiple participants was poor data connectivity and its negative effect on staff technology use. Connectivity issues have been raised as a key barrier in previous research in rural Canadian home care services [30]. These study findings extend this concern about data connectivity issues to the Australian context—in both metropolitan and regional areas.

Furthermore, participants raised several additional important ethical issues related to technology use within in-home aged care, such as client technology preferences and trust of technology. While additional research is required on staff perspectives of ethical issues, the findings align with those from a scoping review of ethical issues related to technology use within aged care, which suggests that ethical concerns, such as lack of regulatory oversight, client autonomy, and nonmaleficence, are important topics for older Australians and their carers [31]. Research also highlights that ethical issues are particularly complex when the aged care recipient lacks the capacity to make an informed decision [9]. Although technology use can offer multiple potential benefits as identified by the study findings, it is paramount for in-home aged care service providers to thoroughly consider the ethical concerns of using technology with care recipients, and that staff are well versed in how to navigate these concerns with care recipients.

### Limitations and Strengths

There were a few study limitations that should be considered when interpreting the findings. First, both the survey and the interviews were conducted using digital platforms. This may have potentially excluded in-home aged care staff members who may feel less comfortable using technology and thus affecting the generalizability and transferability of the findings. However, the digital platforms used for this study are commonly used for a range of operational and administrative purposes within the organization and most staff should be familiar with them. Second, while the demographic characteristics of the staff survey participants were similar to the overall Australian in-home aged care workforce [32], nursing staff were underrepresented. In addition, there was a 12% missing response rate in questions related to enablers and barriers for technology use. This may have limited the generalizability of the quantitative results. Furthermore, interview participants were recruited in 2 ways, with interested staff members self-nominating for the interviews or being nominated by their managers. This may have biased the results of the qualitative interviews. Moreover, the terminology used for direct care tasks may have been interpreted differently by participants. However, the list of direct care tasks was generated through the mapping of organizational service delivery documents and should be relatively familiar and easy to understand for participants. Finally, there were technical issues with the audio recordings of the staff interviews. Due to a storage error, the audio recordings were deleted prior to the completion of data analysis, meaning that the research team was required to rely on the initial transcripts of the interviews. Nonetheless, a strength of the study was the use of a sequential explanatory mixed methods approach, which allowed for an in-depth exploration of the quantitative results [20].

This is the first study specifically investigating Australian in-home aged care workers’ perspectives of the digital enablement potential of direct care tasks, benefits and drawbacks of technology use, and enablers and barriers of technology use. The results of the study would be beneficial for both technology developers and in-home aged care providers, providing key information to guide the implementation of technology into the care provided to older people. As previous literature has pointed out, the importance of understanding end users’ needs and requirements must be prioritized when developing technology for aged care [33]. Our findings will inform technology developers of the relevant tasks and benefits for novel technology and barriers that should be overcome when designing technology for in-home aged care. Previous research has identified that technology implementation is complex and multifaceted in residential aged care, with staff members being a crucial part of the implementation process [34]. This study will allow in-home aged care providers to gain a better understanding of workers’ views on technology use and inform their future technology implementation efforts. Larger-scale studies of multiple aged care providers are required to validate the study findings. Future research should focus on ascertaining evidence-based strategies to facilitate the enablers and overcome the barriers of technology use within Australian in-home aged care.

### Conclusions

This study provides insight into staff members’ views of the digital enablement potential of direct care tasks, benefits and drawbacks of technology use, and the enablers and barriers to technology use within Australian in-home aged care. The study findings would be beneficial for both technology developers and in-home aged care providers, providing key information to guide the implementation of technology into the care provided to older people. Further research is required to ensure that appropriate strategies are available to facilitate the enablers and overcome the barriers to ensure successful implementation of technology into in-home aged care.

## Supplementary material

10.2196/76141 Multimedia Appendix 1 Interview guide and materials.
