# Supplementary material for: The Use of Technology to Deliver In-Home Aged Care Services: Mixed Methods Study of Australian Staff Perspectives
Source: JMIR Aging. 2025 Oct 14;8:e76141. doi: 10.2196/76141 (PMC12520620; doi:10.2196/76141)
Supplement: Multimedia Appendix 1 [file aging-v8-e76141-s001.docx]

## Multimedia Appendix 1. Interview guide and materials.

Interview guide for phase 2 semistructured interviews:

**Sample script at the start of the interview:**

Thank you for agreeing to participate in the interview. Before we start, I would like to let you know we will be asking questions around your views of using technology in home-based aged care. You may think of examples from your work when answering these questions. Please try to avoid providing any client-identifiable information if possible. However, please don’t worry if you do, as we will be removing this information during the transcribing phase to protect the privacy of our clients. Hence, no potentially client-identifiable information will be provided to the research team members not involved in conducting the interview and external to your organisation.

**Interview questions:**

Introduction

Can you tell me a little bit about your role in your organisation?
*What is your role at your organisation?
How long have you worked in this role for your organisation? Overall for your organisation? In the sector?
What area do you work in (e.g. metro/regional/remote)?*

Technology use

What kind of technology or digital services do you use on a regular basis?
*Could include computers/laptops, smartphones, wearables, smart devices (e.g. smart speakers, smart scales etc…)*
*In your personal life?
As part of your role at your organisation?*

Future technology use

What are your thoughts about introducing more technology into your role?
*Do you think it will help you in your role? Why/why not? In what way?
Do you think it will make your role harder? Why/why not? In what way?*

Tasks that could be digitally enabled

As you know, you completed a survey with staff about their perspectives on this topic. Some of the responses from the survey related to which different tasks could be assisted by technology ranked by likelihood include…(Refer to Appendix 1 below).
What do you think about this list? *Do you agree? Disagree?
Are there any activities you would add to this list? Are there any that you would remove? Why?*

Enablers and barriers of digital enablement of care tasks

In the survey we also asked staff about the barriers and enablers of introducing technology into home-based aged care. Some of the responses from the survey of what they said ranked by importance include ………………...(Refer to Appendix 2).

What do you think about this list? *Do you agree? Disagree?
Are there any barriers or enablers that you would add to this list? Are there any you would remove? Why?*

*There are a few more that have been proposed by staff. Where would you put them on this list?*

*Can you tell me a little bit more about why these factors may help or hinder using technology as part of your role?
Can you think of any strategies that may help in overcoming these barriers?*

Technology influencing care quality

How do you think the introduction of more technology into home-based aged care may affect care quality?
*Do you think it will be positive? Negative? Why?
Can you think of any strategies that might lessen any negative effects on care quality?*

Interview materials

Care tasks that may be digitally enabled (Ranked by likelihood)

Managers/Team Leads

| Communicating with team members |
| --- |
| Staff management |
| Onboarding/Training team members |
| Client communication |

Home Care Package Coordinators/Care Managers

| Liaising with other health professionals |
| --- |
| Client assessment |
| Staff management |
| Care coordination |

Domestic Assistants

| Unaccompanied shopping (delivered to home) |
| --- |
| Linen services |
| General house cleaning |

Care Aides/Therapy Assistants

| Assistance with self-administration of medications |
| --- |
| Support with mobilising (e.g. getting in and out of bed/moving around the house) |
| Support with transport |
| Social support |
| Assistance with allied health therapy |
| Support with personal care (e.g. toileting, bathing, dressing, grooming) |

Enrolled Nurses/Registered Nurses

| Nursing Assessment |
| --- |
| Liaising with other health professionals |
| Client education |
| Nursing treatment |

Allied health professionals

| Liaising with other health professionals |
| --- |
| Allied health Assessment |
| Client education |
| Allied health treatment |

Barriers and Enablers

**List of enablers** of introducing technology into home-based aged care (Ranked by importance)

| Having someone to go to if/when the technology does not work |
| --- |
| Having technology that is reliable |
| Having technology that is suitable and appropriate |
| Having good organisational and managerial support |
| Having staff involvement in how technology is implemented |
| Having enough time to get familiar with the technology |
| Having education and training on how to use technology for care tasks |
| Your confidence using technology for care tasks |
| Your previous experience in using technology for care tasks |

Additional enablers:

- Having technology that is an improvement to current practice
- Having clients aware of the change/implementation in technology
- Having system interoperability of new technology with existing systems
- Having external infrastructure to support new technology such as network coverage
- Having safe and secure new technology
- Personal readiness for change and/or new technology

**List of barriers** of introducing technology into home-based aged care (From **Staff/Organisation’s point of view**) (Ranked by importance)

| Having unreliable technology |
| --- |
| Having inappropriate or unsuitable technology |
| Having limited or no support when technology does not work |
| Having limited organisational and managerial support |
| Having limited or no staff involvement in how technology is implemented |
| Having limited time to get familiar with the technology |
| Limited education and training on how to use technology for care tasks |
| Cost to client |
| Your confidence using technology for care tasks |
| Concern that introducing technology will increase workload |
| Client ability, familiarity and confidence using technology |
| Your previous experience in using technology for care tasks |
| Client health status |

**List of** **barriers** of introducing technology into home-based aged care (From **client’s point of view**) (Ranked by importance)

| Cost to client |
| --- |
| Client ability, familiarity and confidence using technology |
| Client health status |

Additional barriers:

- Privacy/Confidentiality concerns from staff/clients
- Client’s lack of trust of technology
- Physical concerns related to using technology
- Insufficient staff readiness for change/implementation
- Lack of staff/client motivation for implementation
- Negatively affecting standard of care
- Lack of respect of dignity of risk
- Lack of information for staff during implementation
- Lack of external infrastructure to support new technology such as network coverage
